# Self-Administered Questionnaire Is a Reliable Measure of Coffee Consumption

**DOI:** 10.2188/jea.JE20090189

**Published:** 2010-09-05

**Authors:** Katri Sääksjärvi, Paul Knekt, Satu Männistö, Markku Heliövaara

**Affiliations:** National Institute for Health and Welfare, Helsinki, Finland

**Keywords:** agreement, coffee, lifestyle, repeatability, socioeconomic factors

## Abstract

**Background:**

The objective of this study was to assess the agreement and repeatability of 2 methods of measuring habitual coffee consumption, and to examine their homogeneity with respect to socioeconomic and lifestyle factors.

**Methods:**

Data on coffee consumption were collected from 4254 subjects by means of a health questionnaire (HQ) and a 1-year dietary history interview (DHI), the latter of which was used as the reference method during the Finnish Mobile Clinic Health Examination Survey conducted in 1973–1976. Short-term repeatability of the methods was assessed using data from 286 and 93 subjects who repeated the HQ and the DHI, respectively, after an interval of 4 to 8 months. The strength of agreement between the 2 methods and between the repeated measurements was estimated using intraclass correlation coefficients (ICCs).

**Results:**

The ICC was 0.86 for the agreement between the HQ and the DHI, and 0.77 and 0.85 for the repeatability of the HQ and the DHI, respectively. There were no statistically significant systematic differences in mean intake values between the 2 methods or between repeated measurements. In subgroup analysis of background variables, there were only minor differences in agreement and repeatability, with somewhat higher ICC values among subjects with a healthier lifestyle and higher education.

**Conclusions:**

The high reliability and homogeneity of the health questionnaire make it a useful tool for measuring habitual coffee consumption for the purposes of epidemiological research.

## INTRODUCTION

Coffee consumption has been associated with several health-related conditions. The drinking of unfiltered coffee has been shown to increase serum levels of total and LDL cholesterol.^1^ Consumption of food items containing caffeine, mainly coffee, has also been associated with an increased risk of spontaneous abortion.^2^^,^^3^ Furthermore, it has been suggested that coffee consumption might be a risk factor for rheumatoid arthritis.^4^ However, coffee consumption may be inversely associated with the incidence of liver cirrhosis, type 2 diabetes mellitus, and Parkinson’s disease.^3^

Coffee consumption has often been measured by items on dietary questionnaires and interviews.^4^^–^^8^ However, because dietary questionnaires have limitations, it is necessary to assess repeatability and to compare questionnaires with another dietary assessment method. With respect to measuring coffee consumption, studies have noted satisfactory repeatability and high overall agreement between 2 methods.^6^^,^^8^^–^^10^ Further quality issues have not been suitably addressed, however, including the question of possible systematic differences between 2 measurement occasions (repeatedly with the same or another method)^11^^–^^14^ or the question of consistency of reliability between subgroups of potential effect-modifying factors (eg, age, sex, education, and body mass index).^8^^,^^13^^–^^16^ Furthermore, there is no information on reliability of coffee consumption in Finland in the early 1970s. Such information is, however, of special interest because the Finnish Mobile Clinic Health Examination Survey provides an excellent foundation for epidemiologic follow-up studies of associations between coffee consumption and morbidity.

The present study, which is based on the Finnish Mobile Clinic Health Examination Survey, uses a 1-year dietary history interview as the reference method to investigate whether coffee consumption can be reliably measured with a health questionnaire. In particular, we examined the agreement between methods, the repeatability of both methods, the existence of a possible systematic difference between the measurement occasions, and the homogeneity of reliability between categories of potential effect-modifying factors.

## METHODS

### Study design and subjects

The study populations were drawn from the Finnish Mobile Clinic Health Examination Survey conducted in 1973–1976.^17^ All, or a random sample of, the residents of each of 12 municipalities in 4 regions of Finland (southwest, central, east, and northwest) were invited to participate in the survey. A total of 19 518 men and women aged 20 years or older participated (83% of those invited). Details of the selection process and the characteristics of the population examined have been described elsewhere.^17^^–^^19^

At baseline, all subjects (*n* = 19 518) completed a mailed self-administered health questionnaire (HQ) and returned it at the health examination. The HQ was checked and completed, when necessary, by a trained nurse. Dietary data were collected for 1 in 6, or 1 in 4, of the randomly selected subjects, and a total of 4343 dietary history interviews (DHI) were satisfactorily completed.^20^ There was an interval of 1 to 2 weeks between the administration of the HQ and DHI. Short-term (4–8 months) repeatability of the methods was assessed as part of the study, after selected subjects repeated the HQ (*n* = 286) and the DHI (*n* = 93).

Subjects with missing information on any background variable were excluded. As a result, 89, 4, and 1 of the subjects were excluded in the comparison of the HQ and the DHI, the repetition of the HQ, and the repetition of the DHI, respectively. Thus, the present study consisted of 3 populations: 1) subjects who completed the HQ and participated in the DHI (*n* = 4254), 2) those who completed repeated HQs (*n* = 282), and 3) those who participated in repeated DHIs (*n* = 92).

### Data collection and dietary and background variables

Both the HQ and the DHI provided information on coffee consumption. On the HQ, habitual coffee consumption was assessed with an open-ended question asking about the average number of cups drunk per day. These were converted to intake in grams per day using 110 g as the volume of a cup of coffee, which was considered a common cup size at the time. A structured DHI was used to collect data on habitual food consumption during the previous year, and was conducted by trained study personnel.^20^ Coffee consumption was inquired about in 2 parts of the interview, as 1) food items used at breakfast and 2) coffee consumption during the rest of the day. The amount of coffee consumed was estimated in grams; cups and glasses of different sizes were used to assist in estimating intake.

In addition, the HQ requested information on the participants’ socioeconomic background (eg, education and marital status), smoking, alcohol consumption, and physical activity.^19^ All baseline examinations were performed at the mobile clinic. Height and weight were measured and body mass index (kg/m^2^) was calculated. Blood pressure was measured in a sitting position after a 5-minute rest, using a semi-automatic device (Elag BPM-A).^18^ Serum samples were collected, and the cholesterol concentration was determined by an autoanalyzer modification (Auto-Analyzer Methodology N-24a and N-77; Technicon, Tarrytown, NY) of the Liebermann–Burchard reaction.^21^

### Statistical analyses

The intraclass correlation coefficient (ICC), estimated as a reliability coefficient,^22^ was used to measure the strength of agreement. Coffee consumption was included as a continuous variable (grams/day) in the analyses. A test for the difference between ICCs was carried out using Fisher’s r-to-z transformation^23^; however, differences between ICC values were considered relevant only when they were >10%. The statistical significance of the difference between the mean consumption levels of the 2 methods/repeated measurements was assessed with the paired *t* test. The analyses were carried out using SAS software version 9.1 (SAS Institute Inc, Cary, NC, USA 2007).

## RESULTS

### Study populations

Overall, the 3 examined populations did not differ noticeably (Table 1): the average coffee consumption was approximately 6 cups per day. However, the population who underwent repeated dietary history interviews (*n* = 92) had the highest proportion of subjects with low education and the lowest proportion of those with hypertension. In the population in which agreement of methods was studied (*n* = 4254), subjects were less likely to smoke, to take part in leisure-time physical activities, and to live in urban areas than those in the other 2 populations.

**Table 1.** Characteristics of subjects in 3 populations of the Finnish Mobile Clinic Health Examination Survey

Variables	Populations		

	HQ vs DHI^a^	Repeated HQs	Repeated DHIs
	*n* = 4254	*n* = 282	*n* = 92
Age (years) (SD)	50.3 (9.41)	48.9 (8.24)	49.4 (8.38)
Males (%)	51.6	54.3	54.4
Married (%)	79.0	82.3	80.4
Education ≥10 years (%)	26.2	22.4	18.5
Community density, urban (%)	72.2	80.1	78.3
Coffee consumption (cups/day) (SD)	5.70 (2.88)	6.08 (2.70)	6.12 (3.60)
Alcohol consumption (ethanol grams/day) (SD)	5.30 (10.31)	4.97 (8.99)	5.59 (8.40)
Body mass index (kg/m^2^) (SD)	26.1 (3.91)	25.9 (3.73)	25.8 (3.38)
Physical activity ≥3 hours/week^b^ (%)	10.4	13.5	12.0
Smoking, current (%)	25.0	28.0	29.4
Hypertension, yes (%)	25.5	25.9	21.7
Serum cholesterol (mmol/l) (SD)	7.14 (1.43)	7.21 (1.41)	7.02 (1.26)

^a^Agreement between the health questionnaire (HQ) and dietary history interview (DHI).

^b^Leisure-time physical activity (heavy, ≥3 hours/week).

### Main results regarding the agreement and repeatability of the methods

The ICC for the strength of agreement between the HQ and the DHI was 0.86 (Table 2). There was no statistically significant difference between the mean consumption levels (in grams) of the 2 methods (*P* = 0.97). The ICC was 0.77 for the repeatability of the HQ and 0.85 for that of the DHI. There were no statistically significant differences between the mean consumption levels (in grams) of the 2 HQs or the 2 DHIs (*P* = 0.81 and 0.16, respectively).

**Table 2.** Results of analysis of agreement and repeatability in the 3 populations assessed

	*n*	*r*^a^	Average coffee consumption (grams/day)	diff.^b^ (%)	*P*^c^
Agreement between HQ^d^ and DHI^d^	4254	0.86	627^e^	−0.01^f^	0.97
Repeatability of HQ	282	0.77	669^g^ (6.08 cups/day)	0.5^h^	0.81
Repeatability of DHI	92	0.85	673^i^	4.6^j^	0.16

^a^Intraclass correlation coefficient, coffee consumption measured as a continuous variable (grams/day).

^b^Differences between measurements.

^c^Statistical significance of the difference between measurements.

^d^HQ, health questionnaire; DHI, dietary history interview.

^e^From dietary history interview.

^f^Negative value indicates that reported coffee consumption was higher on the health questionnaire.

^g^From first health questionnaire, converted from cups/day to grams/day by multiplying by 110 grams (ie, the estimated average for 1 cup of coffee).

^h^Positive value indicates that reported coffee consumption was higher on the first health questionnaire.

^i^From first dietary history interview.

^j^Positive value indicates that reported coffee consumption was higher on the first dietary history interview.

### Analysis stratified by background variables

#### Agreement between methods by background variables

For the majority of background variables, agreement between subgroups was similar to that found in the total study population: ICCs varied from 0.81 to 0.89 (Table 3). Only for age and physical activity were the differences between subgroups large enough (>10%) to be considered relevant. Agreement was exceptionally low among subjects aged 20 to 29 years (ICC, 0.36). There was an obvious trend in the correlation for subgroups of physical activity: the more subjects exercised, the higher were the ICCs (*P* < 0.001 for comparison of ICCs in any subgroups of physical activity).

**Table 3.** Subgroup analyses: agreement between the health questionnaire and dietary history interview (*n* = 4254)

	*n*	*r*^a^	DHI^b^ (grams/day)	diff.^c^ (%)	*P*^d^
Age (years)					
20–29	26	0.36	722	8.0	0.45
30–59	3379	0.87	641	0.4	0.41
≥60	849	0.80	570	−2.1	0.06
Sex					
Male	2194	0.85	641	0.7	0.31
Female	2060	0.88	612	−0.8	0.14
Marital status					
Married	3360	0.86	635	0.006	0.99
Living alone^e^	894	0.87	598	−0.1	0.92
Education (years)					
1–9	3140	0.85	643	−0.4	0.44
≥10	1114	0.89	583	1.1	0.16
Community density					
Rural	1182	0.84	645	−2.3	0.006
Urban	3072	0.87	620	0.9	0.07
Alcohol consumption (grams/day)					
0	1773	0.86	617	−1.4	0.02
<12	1852	0.88	645	0.8	0.17
≥12	629	0.82	605	1.5	0.26
Body mass index (kg/m^2^)					
<25	1774	0.85	626	−0.5	0.49
25–29.9	1841	0.88	629	0.4	0.54
≥30	639	0.84	626	0.1	0.90
Physical activity^f^					
None	1079	0.82	650	0.3	0.75
Light activity ≥4 hours/week	2734	0.86	625	−0.2	0.71
Heavy activity ≥3 hours/week	410	0.92	589	0.3	0.80
Heavy activity almost daily	31	0.98	536	0.7	0.77
Smoking					
Never	2332	0.89	584	−0.5	0.27
Past	860	0.85	619	0.4	0.70
Current	1062	0.81	730	0.6	0.50
Hypertension					
No	3170	0.86	639	0.2	0.74
Yes	1084	0.86	593	−0.6	0.48
Serum cholesterol					
<7.008 mmol/l^g^	2123	0.87	610	0.1	0.82
≥7.008 mmol/l	2131	0.86	645	−0.2	0.78

^a^Intraclass correlation coefficient, coffee consumption measured as a continuous variable grams/day.

^b^Dietary history interview, average daily coffee consumption in grams.

^c^Differences between the health questionnaire and dietary history interview (%), where negative values indicate that reported coffee consumption was higher on the health questionnaire.

^d^Statistical significance of the difference between the 2 methods.

^e^Single, divorced, or widow/er.

^f^Leisure-time physical activity.

^g^7.008 mmol/l was the median serum cholesterol level of the subjects.

In general, no important systematic differences were found in the mean intake values for the 2 methods.

#### Repeatability of the health questionnaire by background variables

Most background variables did not substantially alter the repeatability of the HQ. The ICCs ranged from 0.67 to 0.93 in the analyzed subgroups (Table 4). Statistically significant differences between subgroups were found for education, physical activity, smoking, and hypertension. Subjects who had at least 10 years of education had a higher ICC than did less-educated subjects (*P* = 0.02). Among subjects who did heavy physical activity for at least 3 hours per week, the correlation was higher than among those who reported light or no physical activity (*P* < 0.001 for all comparisons), and the correlation seemed to increase systematically with increasing physical activity. Never smokers had a higher ICC than did current smokers (*P* = 0.02), and the correlation seemed to systematically decrease in smoking subgroups, ie, the highest ICC was for never smokers. Furthermore, the correlation was lower among subjects with hypertension than among those without it (*P* = 0.04).

**Table 4.** Subgroup analyses: repeatability of the health questionnaire (*n* = 286)

	*n*	*r*^a^	1. HQ^b^ cups/day	diff.^c^ (%)	*P*^d^
Age (years)					
20–29	—	—	—	—	—
30–59	242	0.78	6.03	0.01	0.59
≥60	40	0.67	6.35	−3.1	0.60
Sex					
Male	153	0.76	5.97	−1.3	0.66
Female	129	0.78	6.21	2.6	0.27
Marital status					
Married	232	0.76	6.11	−1.0	0.62
Living alone^e^	50	0.78	5.92	7.8	0.08
Education (years)					
1–9	218	0.74	6.29	0.6	0.77
≥10	63	0.86	5.41	0	1.00
Community density					
Rural	56	0.82	6.25	2.9	0.48
Urban	226	0.75	6.04	1.3	0.55
Alcohol consumption (grams/day)					
0	111	0.81	6.45	1.2	0.63
<12	131	0.75	5.96	0	1.00
≥12	40	0.70	5.43	−0.5	0.95
Body mass index (kg/m^2^)					
<25	136	0.78	6.15	1.3	0.64
25–29.9	105	0.75	5.66	−0.9	0.80
≥30	41	0.70	6.90	0.7	0.87
Physical activity^f^					
None	67	0.69	5.96	3.2	0.48
Light activity ≥4 hours/week	177	0.76	6.17	0	1.00
Heavy activity ≥3 hours/week	35	0.93	5.74	−3.0	0.34
Heavy activity almost daily	3	—	—	—	—
Smoking					
Never	139	0.83	5.91	4.1	0.06
Past	64	0.79	6.00	−2.3	0.53
Current	79	0.69	6.43	−3.1	0.51
Hypertension					
No	209	0.80	6.11	0.5	0.82
Yes	73	0.67	6.00	0.5	0.92
Serum cholesterol					
<7.23 mmol/l^g^	141	0.77	5.96	−2.2	0.41
≥7.23 mmol/l	141	0.76	6.20	3.1	0.25

^a^Intraclass correlation coefficient, coffee consumption measured as a continuous variable cups/day.

^b^The first health questionnaire, average daily coffee consumption in cups.

^c^Differences between the first and second health questionnaires (%), where negative values indicate that reported coffee consumption was higher on the second health questionnaire.

^d^Statistical significance of the difference between the 2 measurements.

^e^Single, divorced or widow/er.

^f^Leisure-time physical activity.

^g^7.23 mmol/l was the median serum cholesterol level of the subjects.

There were no statistically significant differences between the mean consumption levels of the repeated HQs in any background variable subgroup.

## DISCUSSION

### Main results in the total study sample

In the present study, agreement between the HQ and DHI was good (ICC=0.86). This accords with the findings of previous Finnish studies, which reported Pearson’s correlation coefficients of 0.97, 0.90, and 0.72 for the agreement between coffee consumption measured by a food frequency questionnaire and 10-, 14-, and 28-day dietary records, respectively.^5^^,^^9^^,^^10^ In general, studies of the agreement between different methods of measuring coffee consumption have reported correlation coefficients ranging from 0.69 to 0.83.^6^^,^^8^^,^^24^ Most of these studies compared a food frequency questionnaire against multiple 7-day dietary records and used Pearson’s correlation coefficient to assess the agreement. It has been shown that regularly eaten foods tend to have higher correlation coefficients.^6^ In Finland, 82% of adults consume coffee daily,^25^ with one of the highest per capita consumption rates in the world.^26^

In the present study, the repeatability of both methods at an interval of 4 to 8 months was good: the ICC was 0.77 for the HQ and 0.85 for the DHI. Accordingly, studies of the repeatability of food frequency questionnaires or dietary interviews with intervals of 1 to 18 months have reported correlation coefficients for coffee consumption ranging from 0.71 to 0.92.^4^^,^^6^^,^^8^^–^^10^^,^^27^^–^^29^

No systematic differences in the amount of coffee consumed were found when measured by the HQ or the DHI, or by repeated measurements with the same method. Only a few studies have described possible bias between 2 measurements of coffee consumption; however, the results of these studies were inconsistent, as some noted a systematic difference,^11^^–^^14^ while others did not.^13^^,^^30^

### Subgroup analyses by background variables

In general, the agreement between the 2 methods of measuring coffee consumption was similar in subgroups analyzed by background variable (ie, age, sex, marital status, education, community density, alcohol consumption, body mass index, physical activity, smoking, hypertension, and serum cholesterol). The only exceptions were age and physical activity. Agreement was considerably weaker among individuals aged 20 to 29 years than among older age groups. This finding may be due to chance, because the number of cases in that group was small. It is also possible that younger subjects were less accurate in their answers, due to lack of interest in health surveys or more irregular dietary habits.

The repeatability of the health questionnaire was, in general, quite similar among all investigated subgroups. There were, however, slight differences between groups classified by education, physical activity, smoking, and hypertension, implying that lifestyle may be related to the repeatability of the method. Subjects who had less education, were less physically active, or were smokers had lower agreement between HQs than did more highly educated individuals and those with healthier lifestyles. This may be because individuals with a healthier lifestyle are more likely to be concerned about their health and thus are more willing to take part in surveys regarding diet and health. They might also provide more accurate estimates of their dietary intake because, as part of their healthier lifestyle habits, they may be more conscious of their diet.

In line with previous studies of coffee consumption, we did not find a sex difference in the agreement between the 2 methods^13^^,^^14^^,^^16^ or in their repeatability.^15^ Reliability studies of coffee consumption that examined age and education subgroups have had contradictory results.^8^^,^^14^^,^^15^ We observed higher repeatability among more educated subjects, but the agreement between the 2 methods was not notably affected by education.

### Methodological considerations

There are several advantages of this study. First, the large size of the study population led to stable estimates and enabled us to conduct subgroup analyses. Second, both the agreement between methods and their repeatability could be evaluated. Third, because information on socioeconomic factors and lifestyle was available, the consistency of reliability could be studied. Fourth, underreporting was likely minimal, because coffee consumption is socially acceptable and prevalent in Finland. Fifth, the type of coffee consumed was likely quite homogeneous, as unfiltered boiled coffee was presumably the predominant method of brewing coffee at the time of this survey.

There are, however, some methodological shortcomings that warrant consideration. First, the repeatability of the DHI could not be examined in the subgroups analysis due to the small size of that population. In addition, the small number of participants aged 20 to 29 years impeded examination of that age group in all analyses. Second, the health questionnaire was not originally designed for measuring dietary data. However, it included an item requesting information on coffee consumption as number of coffee cups consumed per day; many food frequency questionnaires gather the same type of information.

Third, the health questionnaire did not inquire about the portion size of coffee cups consumed or the strength of the coffee brewed. However, agreement between the methods was excellent when information on the number of coffee cups per day was compared with the amount of coffee estimated as grams per day. This suggests that the individual portion size consumed by the participants was very similar between the 2 measurement occasions, and thus the question of the size of the coffee cup is not a major limitation. Lack of information on the strength and type of the coffee consumed does not affect the agreement or the repeatability of the method, but it has to be taken into account in the study of associations between coffee consumption and disease outcomes.

Fourth, the dietary history interview may not be the ideal reference method because the sources of errors may correlate with the health questionnaire (for example, reliance upon memory and conceptualization of portion sizes). Dietary records have traditionally been regarded as the gold standard for examining the reliability of a food frequency questionnaire.^31^ However, as all dietary methods include some level of inaccuracy, comparison of methods can only indicate the degree of agreement between the methods, not the true validity. Nonetheless, even if the correlation coefficient for the agreement between the health questionnaire and the dietary history interview was overestimated, we believe that the results describe the agreement relatively well. In addition, a dietary history interview assessing habitual food consumption during the previous year accounts for variation in diet and thus describes the general diet better than dietary records limited to a few days, assuming the dietary records were not repeatedly kept throughout the year.

Finally, there are some methodological issues to be considered in the interpretation of the results. First, a comparison of present and past results should take into account the fact that ICCs tend to yield slightly lower values than do Pearson’s correlation coefficients. However, they both assess agreement at approximately the same level. Second, since the comparison of 2 different methods includes both the variation between and within the methods, one would expect a repetition of the same method to yield higher ICC values than a comparison of 2 different methods. However, in our study, the agreement between the 2 different methods showed a higher correlation than did repeated measurements of the methods. This was likely due to the fact that there was an interval of 4 to 8 months between the repeated measurements, but only an interval of 1 to 2 weeks between the administration of the HQ and DHI. The check-up for the HQ was actually conducted on the same day as the DHI, which in some respects explains the exceptionally high agreement. Third, generalization of the results of this study requires great care, as coffee consumption habits vary substantially from one country to another. Presumably, the results for Finland apply relatively well to countries with similar coffee consumption habits, ie, countries with a high intake and rather homogeneous consumption habits.

In this study, both methods assessed habitual coffee consumption, which is of interest when studying associations between coffee consumption and disease outcomes. However, the disadvantages of the DHI are that it is expensive, time-consuming, and quite burdensome for the participants. For future epidemiological studies, it is useful to know that a simpler method can provide data of similar quality.

In sum, this study assessed the reliability of a questionnaire for measuring coffee consumption. Both the agreement of the health questionnaire with the reference method and the repeatability of the health questionnaire were good, when ICCs and bias were evaluated. Homogeneity was also high when the agreement between the methods and the repeatability of the health questionnaire were examined, although our study did reveal potential factors related to health behavior (eg, physical activity, smoking, education) that showed a tendency toward an association with the reliability of the questionnaire method. Therefore, we conclude that, for the purposes of epidemiological research, a health questionnaire inquiring about the number of cups of coffee consumed per day is a suitable method for measuring habitual coffee consumption in Finnish adults.
